# Reusable, extensible, and modifiable R scripts and Kepler workflows for comprehensive single set ChIP-seq analysis

**DOI:** 10.1186/s12859-016-1125-3

**Published:** 2016-07-05

**Authors:** Nathan Cormier, Tyler Kolisnik, Mark Bieda

**Affiliations:** Department of Biochemistry and Molecular Biology, University of Calgary Cumming School of Medicine, Rm HSC1151, 3330 Hospital Dr. NW, Calgary, AB T2N4N1 Canada

**Keywords:** Scientific workflows, ChIP-seq analysis, Software packages, Bioconductor

## Abstract

**Background:**

There has been an enormous expansion of use of chromatin immunoprecipitation followed by sequencing (ChIP-seq) technologies. Analysis of large-scale ChIP-seq datasets involves a complex series of steps and production of several specialized graphical outputs. A number of systems have emphasized custom development of ChIP-seq pipelines. These systems are primarily based on custom programming of a single, complex pipeline or supply libraries of modules and do not produce the full range of outputs commonly produced for ChIP-seq datasets. It is desirable to have more comprehensive pipelines, in particular ones addressing common metadata tasks, such as pathway analysis, and pipelines producing standard complex graphical outputs. It is advantageous if these are highly modular systems, available as both turnkey pipelines and individual modules, that are easily comprehensible, modifiable and extensible to allow rapid alteration in response to new analysis developments in this growing area. Furthermore, it is advantageous if these pipelines allow data provenance tracking.

**Results:**

We present a set of 20 ChIP-seq analysis software modules implemented in the Kepler workflow system; most (18/20) were also implemented as standalone, fully functional R scripts. The set consists of four full turnkey pipelines and 16 component modules. The turnkey pipelines in Kepler allow data provenance tracking. Implementation emphasized use of common R packages and widely-used external tools (e.g., MACS for peak finding), along with custom programming. This software presents comprehensive solutions and easily repurposed code blocks for ChIP-seq analysis and pipeline creation. Tasks include mapping raw reads, peakfinding via MACS, summary statistics, peak location statistics, summary plots centered on the transcription start site (TSS), gene ontology, pathway analysis, and de novo motif finding, among others.

**Conclusions:**

These pipelines range from those performing a single task to those performing full analyses of ChIP-seq data. The pipelines are supplied as both Kepler workflows, which allow data provenance tracking, and, in the majority of cases, as standalone R scripts. These pipelines are designed for ease of modification and repurposing.

## Background

Chromatin immunoprecipitation followed by sequencing (ChIP-seq) is a standard approach for localizing proteins bound to DNA, usually transcription factors or histones, including modified histones. The rapidly decreasing cost of sequencing has led to an explosion in the number of ChIP-seq datasets. This is the standard approach used by the large scale ENCODE [1] and modENCODE projects [2].

A comprehensive ChIP-seq analysis is complex and consists of many steps. The steps involved in basic ChIP-seq analysis have been discussed previously [3]. Here, we focus on developing pipelines for analyzing single experiments (optionally with a matched control track). Briefly, first the sequence reads are aligned to a reference genome, then peaks are predicted, and finally a rich analysis of the peak data follows. The analysis of the peaks can be quite complex, encompassing several distinct and independent functions, ranging from motif analysis to pathway analysis. For a full analysis of ChIP-seq data, it is desirable to have a range of outputs from a pipeline, including informative plots to visualize the data.

Generally, ChIP-seq analysis represents an area of complex, multistep data analysis with continuing evolution of analysis options and goals. Under these conditions, the virtues of modifiability, extensibility, and comprehensibility leading to easily reproducible research become important [4]. Modifiability and extensibility are important due to changes in analysis goals (e.g., different types of graphical output) and changes in analysis methodologies or addition of new methodologies (e.g., addition of pathway analyses). With this evolution of pipelines over time, it becomes important to have a software design approach that promotes comprehensibility, because external, written descriptions of functionality can quickly become outdated. Finally, a central scientific value is reproducibility of research results. For complex computational analyses, replication has emerged as a difficult issue for several reasons, as discussed in [5]. Reproducible scientific analyses are supported by systems that feature straightforward distribution of the software and clear display of input values (input parameters). Virtues of various systems to enable reproducible and easily understood analyses have recently been described [6].

There has been significant pipeline development previously in this area and the generation of a large series of tools. Table 1 compares our software to current ChIP-seq analysis packages. This table indicates functions from the perspective of single set ChIP-seq analysis, which is the goal of our pipelines. Importantly, several of the other pipelines provide support for other types of analyses, such as cross-dataset comparisons, cross-species comparisons, ChIP-chip vs ChIP-seq comparisons, and integration of gene expression microarray information. We do not include in the comparison some other pipelines that are oriented toward different tasks, as these pipelines lack most of the functions listed; seqminer [7], according to the authors, is oriented toward analysis based on predefined genomic regions; the ENCODE pipeline [8] includes no downstream analysis; and chipseq [9] includes minimal downstream analysis. We also do not include Sole-Search [10], as this package does not appear to be available currently. Examination of Table 1 indicates that one major difference is that our software provides both complete “turnkey” pipelines and also a set of fully functional independent programs. In contrast, many other systems (e.g., Cistrome) provide only a set of modules, relying on the user to decide on usage and execute them sequentially. A second major difference is that we enable pathway analysis. To do so, we developed a simple scoring scheme for the peaks (see IMPLEMENTATION and RESULTS section for description). We found that pathway analysis provided valuable information in a least one test case. A third significant difference is that our software, like the Cistrome system but unlike the other systems, provides complete “tracking information” (i.e., “data provenance”) in the Kepler implementation. Unlike Cistrome, we supply a set of complete turnkey pipelines that feature data provenance. Being able to determine, after the fact, the exact parameters used in to generate a set of results is considered an important aspect of reproducible science and is a major goal of scientific workflow systems [11]. Finally, as a more minor point, our software provides automatic generation of a user-controllable number of plots of peaks and non-peak regions. Unlike some other packages, although we provide de novo motif discovery functionality, we do not provide motif scanning. We chose to not use this because we viewed it as less valuable and that this basic information is precomputed at other sources (e.g., UCSC genome browser tracks).

**Table 1:** Feature Comparison

Feature	CisGenome [44]	unix tool scripting [45]	Fish the Chips [46]	R pipeline [47]	HiChIP [48]	Cistrome [49]	This package
Primary implementation	C	unix tools	C++	C and R	R	Galaxy	R and Kepler
Single modules available	no	no^a^	no	no	no	yes	yes
Single turnkey pipeline	no	no	yes	no	yes	no	yes
Interface	GUI, command line	command line	GUI, command line	command line	command line	web	GUI (Kepler) + command line
Data provenance tracking	no	no	no	no	no	yes (Galaxy)	yes (Kepler)
Generate genome browser track	yes (custom)	yes (UCSC)	yes (UCSC)	no	yes (UCSC)	yes (UCSC)	yes (UCSC)
Peak calling	yes (custom)	yes (MACS)	yes (MACS)	yes (PICS)	yes (MACS and SICER)	yes (MACS)	yes (MACS)
Summary peak statistics	yes	yes	yes	no	yes	yes	yes
Summary location statistics	yes	yes	yes	no	yes	yes	yes
Map to nearby genes	yes	yes	yes	no	yes	yes	yes
Automatic generation of peak graphs	no	no	no	no	no	no	yes
Heatmap of read density	no	no	no	no	yes	yes	yes
Average profiles	no	no	no	no	yes	yes	yes
De novo motif analysis	yes	yes (MEME)	no	yes (rGADEM)	yes (MEME)	yes (SeqPos)	yes (MEME)
Motif enrichment analysis	yes	yes	no	yes	no	yes	no
Gene ontology	no	yes	no	no	yes(custom)	no^b^	yes
Pathway analysis	no	no	no	no	no	no	yes

^a^Software is a listing of code in a protocol ^b^Gene ontology implemented for microarray data but not ChIP-seq data

We developed a set of 20 workflows: 4 full workflows, 12 independent module workflows, one other independent workflow, and three utility workflows. The great majority of workflows, including full workflows, are available as both standalone R scripts and Kepler workflows (n.b. workflows that rely on external programs will require installation of those programs). All required programs and modules are open source and freely available. Our workflows are initially configured for human datasets. However, many modules do not depend on the organism/assembly (e.g., CalcPeakStats) and others are designed to be easily modified for non-human organisms. For example, organism specific databases are listed as parameters. The full workflows were designed to start from various points in analysis. In some cases, investigators will want to begin with the raw sequence reads. However, in other cases, the investigators may already have aligned reads and, in some cases, predicted peaks for the ChIP-seq data. The independent modules allow the user to execute a single analysis step. We also anticipate that these modules will be reused by other bioinformaticians in their pipelines. We include one independent workflow for data visualization. Finally, the utility set of workflows includes workflows that perform tasks such as simple file manipulation and conversion.

## Implementation and results

Pipelines were based primarily on R, with an emphasis on use of applicable Bioconductor [12] modules and well-established external programs. External programs included bowtie [13] for sequence alignment, MACS [14] for peak determination, MEME [15] for motif discovery, ngs.plot [16] for some visualization, and bedtools [17] and samtools [18] for some file conversion tasks. These pipelines can be easily modified to use other versions of software, for example MACS2 [19] instead of MACS. The modular workflow design enables relatively straightforward complete replacement of these components by other software (e.g., an alternative peak-detection program such as SICER [20]) by the motivated user. We supply a complete archive containing all pipelines and documentation (see “Availability of data and materials” section).

All pipelines were tested using typical, low cost computers, except for read mapping, which was performed using upload to a remote single 32 core (64 thread) Xeon processor machine with 1TB of RAM. However, for ChIP-seq read mapping, a much smaller amount of RAM is generally required [13]. All pipeline components and pipelines (aside from read mapping) easily executed on an 8 Gb RAM, Intel i7 processor based system. The largest full pipeline took ~45 min to execute under these conditions, including read mapping on the remotely located Xeon computer. Disk space was not a significant limitation; 20 Gb free space could easily accommodate a full analysis including initial datasets. We also found that most modules successfully ran on much older computer hardware (3 Gb RAM, Intel Pentium-based desktop computer). We tested the pipelines extensively with a histone modification dataset (H3K4me3 [21, 22]) and a transcription factor dataset (GATA1; [23]).

Workflows were implemented in the workflow development environment Kepler and also as R standalone scripts. The advantages and usage of Kepler in bioinformatics contexts have been previously described [4, 24]. Users who wish to avoid the Kepler environment or simply stay within R can use the provided separate standalone R scripts for most tasks. These R scripts were designed to be relatively short and simple to allow rapid reuse and alteration.

The Kepler workflows can be used without knowledge of programming. The user simply changes the values of the variables which are indicated clearly on the Kepler canvas. Use of the R scripts requires very little additional knowledge. A bioinformatician with minimal R knowledge simply needs to open the scripts in a text editor and edit the parameters, which are all in a block at the start of the code. We suggest that even the non-programmer could be easily taught to open the R file in a text editor, change names, save the R file, and then execute it.

Both full workflows, encompassing the entirety of the analysis tasks, and single components were developed. The full workflows were designed to begin from various established data analysis entry points. Full workflows can begin with unaligned reads, aligned read files, or predicted peaks + aligned reads files. This offers great flexibility for investigators who have precomputed peak sets or aligned reads.

**Fig. 1:**
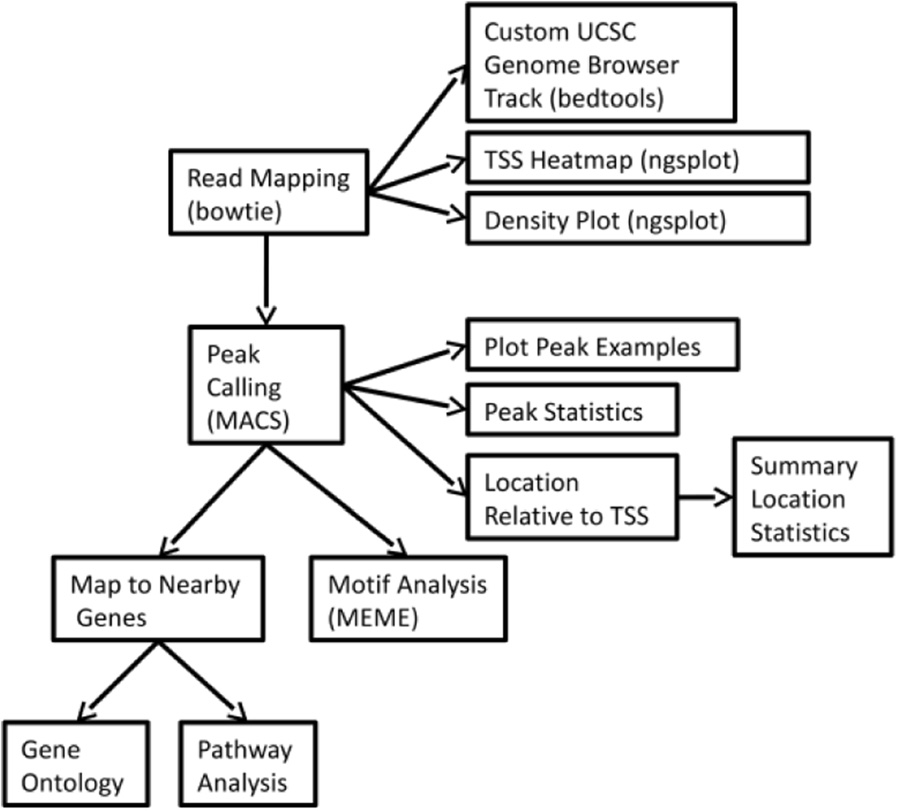
Conceptual diagram of overall project components. Components and relationships for a full analysis of a ChIP-seq dataset. These steps are present in the full workflow. It is provided in Kepler and R script versions (R script version does not have read mapping) and each component is also implemented individually as separate Kepler workflows and R scripts. Names in parentheses refer to critical external, stand-alone programs (e.g., bowtie, MACS, ngsplot, MEME). Most components use R and Bioconductor packages. Some minor steps in the full pipeline (e.g., file conversion), accessory and other utility workflows are not displayed

Figure 1 displays the overall project logic. This figure displays approximate dependencies of outputs on key steps in the pipeline. The boxes on the left display central operations (read mapping, peak prediction, mapping to nearby genes and motif analysis); the boxes to the right display derived information based on these key operations. This figure conveys the essential sets of operations performed; utility operations like file conversion are not indicated.

Table 2 displays a listing of the full set of workflows and a description for each workflow. R scripts, when available, are also indicated. We use the notation “name(.kar/.R)” to succinctly indicate that workflow “name” is available as a Kepler workflow (“name.kar”) and as an R script (“name.R”).

**Table 2:** List of Software Modules

Name	Goal
Full Pipelines	
Pipeline_UnmappedReads.kar	Full pipeline starting with unmapped reads
Pipeline_MappedReads(.kar/.R)	Full pipeline starting with mapped reads
Pipeline_MappedReadsAndExternalPeakFile(.kar/.R)	Full pipeline starting with mapped reads and a file of peaks
Pipeline_onlyExternalPeakFile(.kar/.R)	Full pipeline when only file of peaks is available
Independent Modules	
MapReads.kar	Map reads to a reference genome
AnnotatePeaks(.kar/.R)	Map peaks to genes
CalcDistanceToTSS(.kar/.R)	Summary of peak distance to TSS
CalcPeakStats(.kar/.R)	Summary peak statistics
GeneOntologyAnalysis(.kar/.R)	Gene Ontology Analysis
GeneratePeakExamples(.kar/.R)	Generate a set of graphs of peak and non-peak regions automatically
GetPeakSequences(.kar/.R)	Generate set of DNA sequences from peaks
MakeTSSHeatmapAndDensityPlots(.kar/.R)	Makes a heatmap of reads around all TSSs and also an average profile plot
MakeUCSCfile(.kar/.R)	Makes a bedgraph.tar.gz file ready for direct upload to the UCSC genome browser
MotifDiscovery(.kar/.R)	Runs MEME to attempt motif finding from peaks
PathwayAnalysis(.kar/.R)	Performs a pathway analysis, generates list of high-scoring pathways and images of results
RunMACS(.kar/.R)	Runs MACS to predict peaks
Utility	
BamToBed(.kar/.R)	Converts bam format to bed format
IndexBAM(.kar/.R)	Indexes a bam file
SamToBed(.kar/.R)	Converts sam format to bed format
Extra	
GraphSingleDataRange(.kar/.R)	Generates a graph of read density for any region of the genome

*Notes:* (.kar/.R) refers to workflows having Kepler (.kar) and R (.R) versions. The same root name is used; for example RunMACS.kar and RunMACS.R

**Fig. 2:**
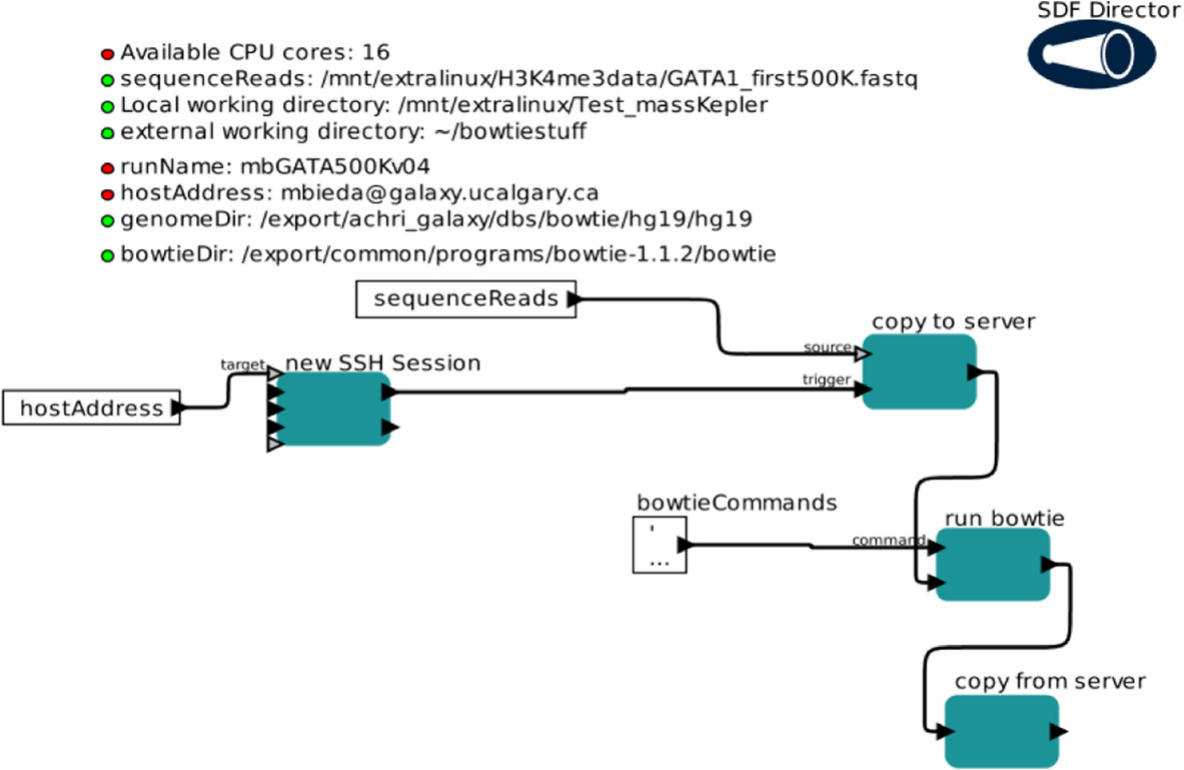
MapReads.kar Kepler workflow. This workflow displays some fundamental features of Kepler and Kepler’s ability to use external computing and data resources. The workflow is executed from the user’s machine but performs mapping of a set of sequence reads on an external high performance machine. A sequence read file (fastq format) is copied to an external machine/cluster and the program bowtie [13] is used to align reads to the genome specified by parameter genomeDirectory. The output file (sam format) is then copied back to the user’s machine. The number of CPU cores used for this computation can be limited by the ‘Available CPU cores’ parameter. This is a screenshot of the actual Kepler canvas that is displayed to the user

One significant advantage of Kepler is straightforward integration of different computational resources, from local resources (i.e., the user’s machine) to remote resources (e.g., a computer cluster). It is advantageous to use high performance computing resources for computationally demanding tasks. For ChIP-seq analysis, aligning raw read sequences to a genome is a computationally demanding task. Figure 2 displays a Kepler workflow for remote alignment of reads. This component is available as a standalone workflow (MapReads.kar) and as part of a full workflow (Pipeline_UnmappedReads.kar). The image shown is a screenshot of MapReads.kar. Note that parameters as input are clearly shown (top part) and the flow of data is shown in the lower part. Parameters are easily changed simply by “clicking” on the appropriate parameter (see [24] for example). The logic of operations is apparent from the workflow: data is copied to the external machine, the process is run, and data is retrieved at completion. This workflow currently is based on bowtie [13] but can be easily modified to use other alignment software. This workflow not only allows usage of a high performance remote computer but also offers a template for shifting other tasks into a high performance external resource. For example, motif finding, a computationally demanding task, would benefit from high performance resources.

**Fig. 3:**
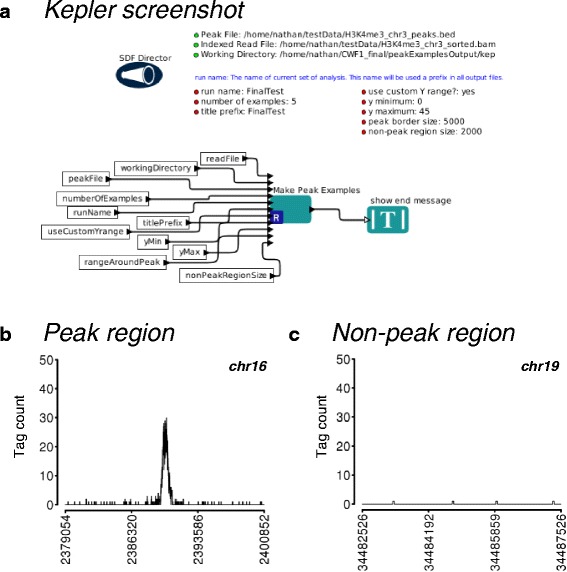
Generation of peak examples. **a** Screenshot of Kepler canvas for this workflow. This workflow shows the typical design for most of our single module Kepler workflows. This task is part of each of the full workflows for ChIP-seq, except those starting with only predicted peaks. It is available as an individual Kepler workflow and as an independent R script. **b**, **c** Output from workflow applied to H3K4me3 dataset (see IMPLEMENTATION AND RESULTS). This workflow produces graphs of positive peaks (example shown in b; x-axis range is chr16:2379054–2400852) and graphs of negative regions (example shown in c; x-axis range is chr19:34482526–34487526). Workflow output graphs are designed for on-screen viewing and graph aesthetics have been adjusted for clarity of presentation here. The number of produced graph examples and axis ranges are controlled by the user. Standard R graphics (“base graphics”) are used for graph generation, allowing the experienced user to easily modify graph properties

Figure 3a displays an example of a more typical Kepler workflow run solely on the user’s computer. We used the same design principle with parameters in the top portion and the logic of dataflow below. Note that nearly all processing occurs in a single R module (“actor” in Kepler workflow terminology).

This workflow produces example graphs of peak regions and non-peak regions (Fig. 3b,c). This is useful for examining the waveforms of high-scoring peaks and producing examples for potential publication as part of a scientific report. For non-peak regions, the workflow searches between peaks for low-coverage regions to display as negative regions (Fig. 3c). Users can adjust parameters such as the number of positive and negative peaks produced and the overall range of the x-axis directly on the Kepler canvas or in the separate, standalone R script. Furthermore, this module uses standard R graphics (“base graphics”), so the motivated user can easily change the code to produce different graph aesthetics.

In all of our standalone R scripts, the parameters are always indicated as block of code to begin the R script. This allows straightforward, clear alterations of parameter values.

**Fig. 4:**
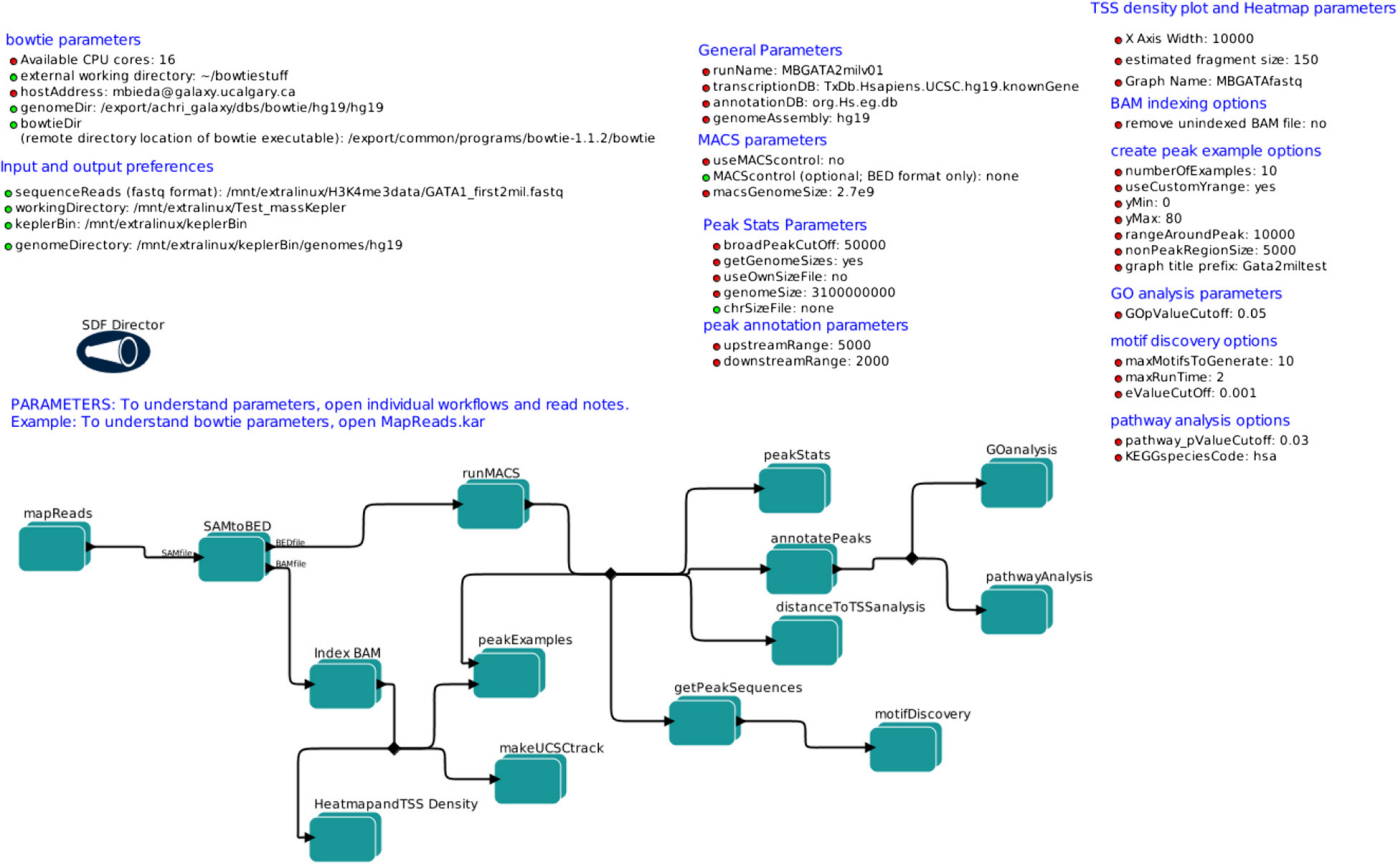
A full analysis pipeline for ChIP-seq data. This displayed workflow begins with raw sequence reads; other variants begin with mapped data or mapped data with called peaks (see Table 2). The workflow implements steps displayed conceptually in Fig. 1. This workflow is also available as a standalone R script (but without initial read mapping step)

Figure 4 displays the full pipeline, as implemented in Kepler (Pipeline_UnmappedReads.kar). This is the “maximal” version of the full pipeline which begins with a file of unmapped sequence reads (a fastq file) as input and then produces a large series of outputs. This pipeline, without the initial read mapping step, is available as a standalone R script (starts with mapped read file; Pipeline_MappedReads.R). For the Kepler version, the parameters are grouped by functional category to allow rapid visual determination of what critical values were used for data processing and analysis. For example, the “General Parameters” section indicates the various files used for mapping peaks to genes. Due to the large number of parameters in the full pipelines, documentation for parameters is included in the R version of this workflow and also in each corresponding independent Kepler module file (e.g., see parameter descriptions in Fig. 3a for GeneratePeakExamples.kar). The motif-finding program MEME [15] has large computational demands and motif finding accounts for the majority of pipeline run time. To control this runtime, we allow the user to limit the MEME runtime using the parameter maxRunTime (units are seconds). MEME produces motifs as it executes, so limiting the runtime limits the accuracy of results but does not prevent MEME from producing results (unless no motifs are found within the timelimit). We found that a GATA1 ChIP-seq dataset yielded a correct GATA1 motif (see below) within a timelimit of 1800 s (30 min) under our conditions.

Investigators will often have a file of already aligned reads and, in some cases, may also have a file of predicted peaks. If the investigators already have these files, it is wasteful to run these slow and computationally demanding steps. Also, the files may have been generated by other programs preferred by the investigators. We wished to create pipelines that allow straightforward usage of these files, when available. For investigators with aligned reads but without predicted peaks, we created a workflow to deal with this situation (Pipeline_MappedReads(.kar/.R)). For investigators with both aligned reads and a file of predicted peaks, we allow investigators to skip the alignment step and the peak prediction step (Pipeline_MappedReadsAndExternalPeakFile(.kar/.R)). Finally, for investigators with only a file of predicted peaks, we supply a workflow for this case (Pipeline_onlyExternalPeakFile(.kar/.R)).

**Fig. 5:**
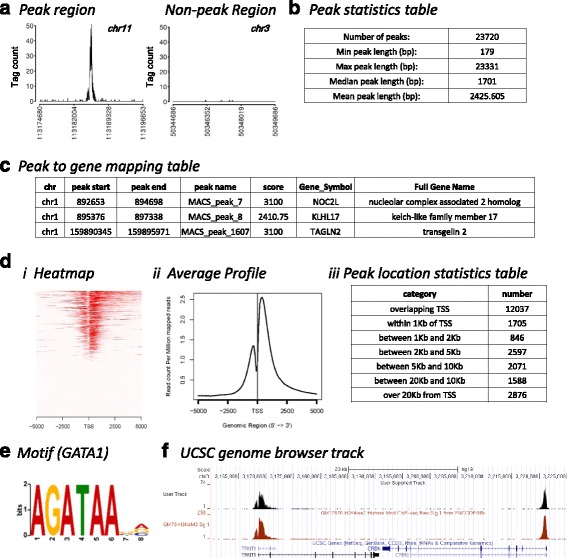
A partial set of outputs of the full pipeline and component workflows. See Fig. 6 for more outputs. All of these are outputs of all the full workflows, except some are not available in the pipeline beginning with only peak data. Each individual output is available as the product of individual workflows or as standalone R scripts (see Table 2). All data is from analysis of the H3K4me3 dataset except E, which is from analysis of the GATA1 dataset. **a** GeneratePeakExamples component output. (*left*) A predicted peak (x-axis: chr11:113174680–113196653) (*right*) A non-peak region (x-axis: chr3:50344686–50349686). Also see Fig. 3. **b** Partial output of CalcPeakStats module. **c** Partial output of mapping to genes workflow (AnnotatePeaks). Several additional columns of information are not shown here and full table has many more rows. **d** (i) Heatmap of coverage around transcription start sites (TSSs). (ii) Density plot of coverage around TSSs. (iii) Table of distances from TSS. Note expected pattern of TSS-centric location of H3K4me3, as indicated in previous studies. **e** Output of motif discovery component for GATA1 dataset. Note close match to expected GATA1 motif [27] is obtained, including central GATA motif (positions 2–5). **f** Comparison of workflow-generated UCSC track (*top*) to ENCODE-generated UCSC track (*middle*) for H3K4me3 dataset. Image from UCSC browser

**Fig. 6:**
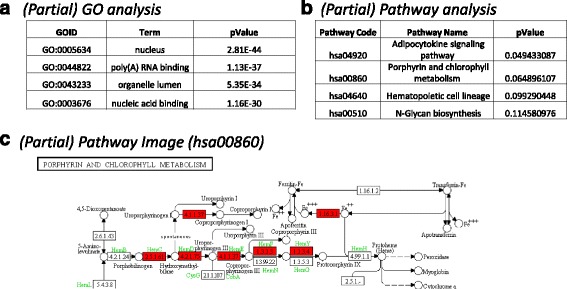
More outputs from the full pipelines and individual components. All outputs are produced from all full pipelines (except the one starting with only peak data), appropriate individual workflows, and Kepler-independent R scripts (see Table 2). Genes possessing a peak in the proximal promoter are made into a list with an associated score equal to score of maximum scoring peak in the proximal promoter. This list is the input into this analysis. **a** Partial gene ontology output for GATA1 dataset. Top four gene ontology categories are shown. **b** Partial GATA1 ChIP-seq pathway analysis results. Top four pathways are shown. **c** Porphyrin pathway (hsa00860) image generated by workflow. Image is truncated for presentation clarity. *Red color* indicates a gene with a peak in the proximal promoter (see IMPLEMENTATION AND RESULTS for details). Genes without any color coding did not have peaks in their promoters. Note that this pathway is strongly implicated in GATA1 function (e.g., [43])

Figures 5 and 6 display the outputs of the full workflows. As discussed above, these are also the outputs of individual workflows and, in most cases, equivalent standalone R scripts (see Table 2). To examine the outputs, we employed a trimethylated lysine 4 of histone H3 (H3K4me3) dataset from the ENCODE project (GM12878 immortalized human B-lymphocytes; sample ENCFF001EXQ [21] and control ENCFF001HHW [22]) and a GATA1 dataset (K562 cells; NCBI GEO GSM1003608; [23]). We show only a portion of rows and columns of displayed output tables to allow reasonable clarity of presentation in figures.

Figure 5a shows the results of generating H3K4me3 peak examples, as in Fig. 3. This workflow is available as part of all full pipelines except those starting with only peak data (see Table 2), as an individual Kepler module and as a standalone R script (GeneratePeakExamples(.kar/.R)).

Figure 5b shows a partial table of the results from computation of basic peak statistics for the H3K4me3 dataset. We created custom functions to calculate these statistics. These results were verified by manual calculation of values from the peak dataset. This workflow is available as part of all full pipelines (see Table 2), as an individual module and as a standalone R script (CalcPeakStats(.kar/.R)).

Figure 5c shows a partial table of the output of assigning peaks to genes. We initially investigated using the Bioconductor package ChIPpeakAnno [25] for this purpose. However, we found that this package creates TSS “ranges” for genes as opposed to having a specific TSS for each transcript. To avoid this issue, we developed our own custom functions. We used the well supported Bioconductor annotation package org.Hs.eg.db and the specific transcript db package TxDb.Hsapiens.UCSC.hg19.knownGene. These are user-adjustable parameters. A wide range of annotation and transcript database packages for other assemblies/organisms are freely available via Bioconductor. Peaks were mapped to genes based on peak presence in the proximal promoter (the user controls the nucleotide range defining the proximal promoter region). For the displayed analysis, a peak was assigned to a gene if the peak is within a range of −5000 nts to +2000 nts of the TSS (where 0 is the location of the TSS). This workflow is available as part of all full pipelines (see Table 2), as an individual module and as a standalone R script (AnnotatePeaks(.kar/.R)).

Figure 5d shows outputs of TSS-centered analyses. Figure 5d(i) shows a “heatmap” of H3K4me3 ChIP-seq reads around the TSS of each transcript. Each row is a single region around a TSS, which is placed at center. Figure 5d(ii) displays the average profile of read density around TSSs. These two functions are available as part of all full pipelines except the peak-only pipeline (see Table 2), as an individual module and as a standalone R script (MakeTSSHeatmapAndDensityPlots(.kar/.R)). Both the heatmap and the density plot are generated by the external program ngs.plot [16]. Figure 5d(iii) shows the output of an analysis of peak location with respect to the TSS by placing peaks into discrete categories based on distance. This component was based on our custom R programming. This workflow is available as part of all full pipelines (see Table 2), as an individual module and as a standalone R script (CalcDistanceToTSS(.kar/.R)). These analysis results accord with previous results for H3K4me3, which is well-known as a TSS-centric epigenetic mark [26]. The “notch” in the center of the mean density graph (Fig. 5d(ii)) is expected due to histone depletion in active genes and has been seen in other studies [26]. Finally, the table of distances also indicates the TSS-centric nature of this epigenetic mark.

Figure 5e displays the top-scoring motif from motif analysis of the GATA1 dataset. The software component is based on the very widely used motif analysis tool MEME [15]. In this case, MEME was limited to running for 1800 s (30 min), which was set by a user-controllable workflow parameter. This displayed motif closely matches an expected motif for GATA1 [27]. This workflow is available as part of all full pipelines beginning with read data, as an individual module and as a standalone R script (MotifDiscovery(.kar/.R)).

Figure 5f shows the results of creating a file for upload to the UCSC browser. The computation for this component employs bedtools [17]. A bedgraph.tar.gz file is produced that can be directly uploaded to the UCSC genome browser [28] for display. Figure 5f shows the ENCODE project results (top) and our computed results (middle). Visual inspection did not reveal large differences in waveforms. This workflow is available as part of all full pipelines except peak-only pipelines, as an individual module and as a standalone R script (MakeUCSCfile(.kar/.R)).

Figure 6 displays the results of gene ontology and pathway analyses for the GATA1 dataset. The inputs for these functions can be lists of genes or the full outputs of the mapping peaks to genes pipeline (AnnotatePeaks(.kar/.R)).

Figure 6a shows partial results of gene ontology analysis for the GATA1 dataset. Our module was based on the Bioconductor module GOstats [29]. Although the top 10 categories revealed only general categories (e.g., GO:0005634 “nucleus”), specific categories related to known GATA1 roles [30] were also present farther down in the list (GO:0034101 “erythrocyte homeostasis” and GO:0007346 “regulation of mitotic cell cycle”). The full table output consists of several additional columns and many more rows of data. This workflow is available as part of all full pipelines, as an individual module and as a standalone R script (GeneOntologyAnalysis(.kar/.R)).

Figure 6b, c shows the results of the pathway analyses. We chose to use the Bioconductor packages gage ([31]; for pathway analysis computations) and pathview ([32]; for pathway visualization). The package gage is completely open source and can analyze single replicate experiments, as opposed to some other pathway analysis approaches (discussed in [31]). Both gage and pathview can use pathway databases from several sources, as described in the documentation for these packages.

The underlying gage computations are designed to use fold change data from gene expression experiments. The relationship of ChIP-seq peak magnitude to gene expression is complex and difficult to predict as to strength and direction (i.e., repression or activation). Some transcription factors may be activators, some repressors, and some mixtures of the two [30]. We chose to first determine the list of genes with peaks in the proximal promoters (see below for discussion). Then, we determined the highest scoring peak in the promoter of each of these genes, and assigned the gene the value of that peak. This list of genes (and associated scores) are then submitted to pathway analysis. With only our ChIP-seq data as a source of information, we do not know if transcription of these genes is being activated, repressed, or not changed by the transcription factor being present in the promoters. Due to this ambiguity, the pathways containing these genes may be upregulated, downregulated, or unchanged by the transcription factor binding in the promoters. The pathway software produces lists of pathways that are labeled as “upregulated” and “downregulated”. We suggest that these labels should be ignored and that all pathways that arise from this analysis are considered simply as “potentially up or down regulated”. This implementation of pathway analysis is available as part of all full pipelines (see Table 2), as an individual module and as a standalone R script (PathwayAnalysis(.kar/.R)).

Figure 6b displays a partial listing of the pathway analysis table. We used the KEGG database of pathways, as described in the gage publication [31]. Due to our scoring modifications, we focused on top-ranking pathways as opposed to *p*-values. Notably, the pathways “Porphyrin and chlorophyll metabolism” (hsa00860) and “Hematopoietic cell lineage” (hsa04640) are found. These pathways match the expectations for GATA1 based on previous work [30]. For this software module, the user controls the *p*-value cutoff. We employed *p* < 0.20 to allow pathway analysis to be used for hypothesis generation as opposed to pathway exclusion.

Figure 6c shows a portion of one pathway output image from the workflow (porphyrin pathway (hsa00860)). Due to the assignment of all genes to a relatively high positive value, all genes that have predicted peaks in the proximal promoter are indicated by red backgrounds. In total, our software generates a table of pathways and images of the pathways.

## Discussion

Chromatin immunoprecipitation followed by sequencing (ChIP-seq) is the current standard approach for localizing proteins attached to genomic DNA on a full genome scale. With decreases in the cost of sequencing, this methodology has become very widely used. Hence, a wide variety of laboratories, from the large scale to the small, are using these approaches. A comprehensive analysis of a ChIP-seq dataset involves many steps with many different types of outputs, ranging from simply mapping the sequence reads to performing gene ontology analyses. There is current intensive development of new analytical approaches and tools in this area, producing a need for pipelines that can be easily altered to employ the latest and best software tools. To address these analytical needs, we developed a suite of pipelines and modules to perform a range of analyses, from a full, turnkey analysis to individual analytical tasks. We focused on the software engineering approach of producing highly modular pipelines using well-established and standard approaches (e.g., R scripts employing existing Bioconductor packages). An alternate approach would be to produce a unitary program that uses command line options to control which modules are executed. Because most bioinformaticians are familiar with R and Bioconductor or can easily find information on this completely open-source and well documented framework, our approach enables rapid modification, extension and repurposing of our work. The highly modular aspect also contributes to the ability to quickly comprehend these pipelines. In our view, this is particularly true in the case of the Kepler workflows, whose graphical nature promotes rapid comprehension. As a secondary point, we applied a simple approach to pathway analysis with ChIP-seq datasets that seemed to produce good results in the case of at least one transcription factor (GATA1). We suggest that pathway analysis may be an important tool for understanding roles of poorly characterized transcription factors and should be a standard ChIP-seq analysis component.

We used two software environments to implement the pipelines. We chose to implement pipelines in both Kepler and as simple R scripts. We chose this dual implementation approach to maximize the utility of our work. Kepler has significant advantages over simple scripting. First, the graphical nature of the Kepler canvas makes the flow of data and dependencies of modules on other modules clear. Furthermore, the Kepler canvas (i.e., screen display) allows flexible placement of comments with control of font properties. This feature promotes user comprehension by allowing the workflow designer to highlight certain points and minimize others. Second, the nature of the Kepler canvas strongly promotes a separation of input parameters from data processing steps. This division makes parameters clear. Third, Kepler, via the bioKepler project [33], is developing powerful abilities to directly interface with complex remote computing platforms (e.g., MapReduce; see [34]). As the amount of data in typical bioinformatics analyses increases, this will become more important. The contrasting virtue of the R scripts (employing existing Bioconductor packages) is their familiarity for many bioinformaticians. Many investigators may wish to avoid the Kepler framework and will be more comfortable with straightforward R scripts. These investigators can take advantage of our work without ever learning Kepler.

We employed a set of software design principles. All of these were in service to the greater goal of promoting extensibility, modifiability, and comprehensibility to enable reproducible research. First, we emphasize the use of the R (with Bioconductor packages) framework for processing, except when standalone programs are the accepted standard. R scripting allows great amounts of customization and this environment is widely used in bioinformatics. We expect that most bioinformaticians will want to modify our graphical outputs for their own purposes. Second, we used a modular development approach. Instead of presenting a single, large optimized program, we chose to develop individual modules performing discrete functions which were then combined to form the full pipelines. This design strategy allows relatively easy changes to modules, which we view as important in this rapidly evolving area of investigation. For example, a bioinformatician wishing to use a different pathway analysis software approach can easily modify the pathway analysis module in our pipelines or the individual modules. Third, we emphasized a careful separation of input values (“parameters”) from data processing (“modules”, “actors”, “workflows”) steps. Kepler naturally supports this division with the Kepler canvas. For our R scripts, we developed a standard script format featuring a delineated block of parameters to enable rapid alteration of values. This design approach promotes comprehension - by making the values of parameters obvious - and extensibility and modifiability by separating data processing from parameters.

Our work focuses on analysis of a single ChIP-seq dataset (with an optional accompanying control/input dataset). However, our work can be extended for analysis of datasets with several replicates. For example, other software (e.g., Galaxy components; [35]) may be used to generate a consensus set of peaks. This consensus set of peaks, along with the sequence data from a single replicate, can be used as input into our full pipeline that starts with peaks + sequence data (Pipeline_MappedReadsAndExternalPeakFile(.kar/.R)). Alternatively, the investigator may use their consensus peak set with our single module workflows/scripts for downstream analyses. For example, the investigator may wish to map the consensus peaks to genes and then perform pathway analyses, which can be easily accomplished using our individual workflows/modules. There are clearly other ways in which our software modules could be incorporated into pipelines for analysis of multiple datasets.

Our work can also be used as a basis for ChIP-seq pipelines addressing different types of scientific questions. Some analyses of chromatin involve comparing datasets from multiple different factors (e.g., bivalent promoters with H3K4me3 and H3K27me3 marks, see [36]). Our modules performing basic initial functions (read mapping, peak prediction) would be useful in this scenario. The more downstream meta-analysis functions (e.g., pathway analysis) will also usually be useful in these cases. Other analyses integrate ChIP-seq data with other types of data, most commonly large scale gene expression data (usually microarray or RNA-seq data) [37]. In this case, our full pipelines may become one component in these more complex analyses, with the data integration steps often logically occurring after all the processing in our pipeline is finished. To our knowledge, existing software for these analyses (e.g., [38]) was not designed specifically with a goal of extensibility and modifiability and hence appears to be much more difficult to extend/modify than our software.

Finally, our work can also be easily adapted to address “broad modifications”. The term “broad modification” refers to a transcription factor or histone modification that is found along a large region of a chromosome (often >50kb to megabase length regions; e.g., see [36]). Recently, there has been development of specialized peak-detection software aimed at accurately detecting broad modifications in ChIP-seq datasets [39, 40]. We anticipate that it would be relatively straightforward to replace MACS in the peak finding step with one of these programs, enabling our pipelines to be used for broad peak detection. However, it will also probably be necessary to modify the peak annotation steps to allow a single lengthy peak to be “mapped” to several genes.

There are some relatively obvious ways in which our pipelines could be enhanced. These primarily involve development and subsequent addition of new functional modules. A quality control step could be implemented at an early stage in the pipeline. At the end of the pipeline, a full report of all results could be produced using “literate programming” tools such as knitr [41] or sweave [42].

## Conclusions

Comprehensive ChiP-seq analysis involves a large number of functional steps. In current software implementations, these often involve many individual software packages. Our ChIP-seq pipelines and standalone, fully independent component modules can be used as-is or can be easily modified for other purposes, including recombination or integration into other pipelines. Our software design approach emphasized modifiability, extensibility, and comprehensibility. These ChIP-seq analysis pipelines are implemented in a robust fashion using Kepler and simple R scripting. It is advantageous to have both complete pipelines and individual functional modules.

## Availability of data and materials

Project name: ChIPSeqWorkflows

Project home page: https://goo.gl/IHHYmM

Archived version: ChIPSeqWorkflows1.0.tar.gz

Operating system(s): Linux

Programming language: R (with standard existing Bioconductor packages) primarily; bash scripting, Python

Other requirements: Python 2.7.x; R > =3.1.2

License: GNU General Public License

## Abbreviations

ChIP-seq, chromatin immunoprecipitation followed by sequencing; RNA-seq, RNA isolation followed by sequencing
